# 

**Published:** 1940

**Authors:** 


### The Medical Library of the University of Bristol

WITH WHICH IS INCORPORATED

**The Library of the Bristol Medico-Chirurgical Society.**

*The following donations have been received since the publication of the list in April,* 1940.

July, 1940.

| | |
|---|---|
| American Otological Society (1) .. .. .. | 1 volume. |
| Messrs. J. Baker & Son (2) .. .. .. .. | 1 volume. |
| British Homœopathic Association (3) .. .. .. | 1 volume. |
| British Medical Association (4) .. .. .. .. | 5 volumes. |
| Brompton Hospital for Chest Diseases (5) .. .. | 1 volume. |
| Buenos Aires University (6) .. .. .. .. | 1 volume. |
| Campden Research Station (7) .. .. .. .. | 1 volume. |
| Ciba Limited (8) .. .. .. .. .. .. | 1 volume. |
| Dr. E. A. Codman, Boston, Mass. (9) .. .. | 1 volume. |
| Professor E. W. Hey Groves (10) .. .. .. | 7 volumes. |
| Dr. J. E. Shaw Bequest (11) .. .. .. .. | 2 volumes. |
| Munro Smith Fund (12) .. .. .. .. .. | 1 volume. |
| National Foundation for Infantile Paralysis (13) .. | 1 volume. |

Unbound periodicals have also been received from Professor E. W. Hey Groves, Professor C. Bruce Perry, Dr. G. de M. Rudolf, and Dr. J. Odery Symes.

### THE ONE HUNDRED AND SEVENTY-FIRST LIST OF BOOKS

The figures in round brackets refer to the figures after the names of the donors. The books to which no such figures have been attached have either been bought from the Library Fund or received through the *Journal*.

| | | | |
|---|---|---|---|
| **Behan, R. J.** .. .. .. | *Relation of Trauma to New Growths—Medico-Legal Aspect* .. .. .. .. .. .. .. | (10) | 1939 |
| **Bertwistle, A. P.** .. .. | *Descriptive Atlas of Radiographs* | 4th Ed. (10) | 1939 |
| **Brain, W. R.** .. .. .. | *Diseases of the Nervous System* .. | 2nd Ed. | 1940 |

| | | | |
|---|---|---|---|
| Buxton, St. J. D. (Ed.) | *British Red Cross Society, Notebook for First Aid Courses* | 2nd Ed. | 1940 |
| *Catalogue of the Periodical Publications in the Libraries of the University of Bristol* | | | 1940 |
| Codman, E. A. | *The Shoulder* | (9) | 1934 |
| Cope, V. Z. | *Pioneers in Acute Abdominal Surgery* | | 1939 |
| Couch, J. H. | *Surgery of the Hand* | (10) | 1939 |
| Davis, C. E. | *The Mineral Baths of Bath* | | 1883 |
| De Brun, H. C. W. S. | *Manual of Fractures, Dislocations and Epiphyseal Separations* | (10) | 1939 |
| Devine, Sir H. | *Surgery of the Alimentary Tract* | (10) | 1940 |
| *Festschrift zur 74 Versammlung deutscher Naturforscher* | | (2) | 1902 |
| Filippi, J. de | *Dietetica Argentina para Bacilosos* | (6) | 1935 |
| Frazer, W. M., and Stallybrass, C. O. | *Textbook of Public Health* | 10th Ed. | 1940 |
| Girdlestone, G. R. | *Tuberculosis of Bone and Joint* | | 1940 |
| Groves, E. W. Hey, and Brickdale, J. F. | *Textbook for Nurses* | 6th Ed. | 1940 |
| Gunn, J. A. | *An Introduction to Pharmacology and Therapeutics* | 6th Ed. | 1940 |
| Henderson, D. K., and Gillespie, R. D. | *Textbook of Psychiatry* | 5th Ed. | 1940 |
| Hirst, F., and Adam, W. B. | *Hydrogen Swells in Canned Fruit* | (7) | 1937 |
| Martin, C. R. A. | *Practical Food Inspection* | 2nd Ed., 2 Vols. | 1940 |
| Miller, J. A., and Wallgren, A. | *Pulmonary Tuberculosis in Adults and Children* | (12) | 1939 |
| Roberts, J. A. F. | *An Introduction to Medical Genetics* | | 1940 |
| Roberts, W. E. | *Surface Anatomy* | (10) | 1937 |
| Schaeffer, M., and Muckenfuss, R. S. | *Experimental Poliomyelitis* | (13) | 1940 |
| Semon, H. C. G., and Moritz, A. | *Atlas of Commoner Skin Diseases* | 2nd Ed. | 1940 |
| *Sex Hormones, The* | | 2nd Ed. (8) | 1940 |
| Thomson, A., and Miles, A. | *Manual of Surgery* | 9th Ed. | 1939 |
| Vallese, T. | *Un ignoto ricettario medico inglese del xiv. secolo* | | 1940 |
| Wheeler, C. E. | *Introduction to the Principles and Practice of Homœopathy* | 2nd Ed. (3) | 1940 |
| Whitnall, S. E. | *Study of Anatomy* | 4th Ed. (10) | 1939 |
| Whittaker, C. R. | *Anatomy. Part III. (Head and Neck).* | 5th Ed. | 1940 |
| Wilson, S. A. K. | *Neurology* | 2 Vols. (11) | 1940 |

### TRANSACTIONS, REPORTS, JOURNALS, ETC.

| | | |
|---|---|---|
| British Medical Association Handbook, 1931–2, 1933–4, 1936–7, 1937–8, 1938–9 | (4) | |
| Brompton Hospital Reports, Vol. VIII. | (5) | 1939 |
| Medical Annual, 1940 | | 1940 |
| Quarterly Cumulative Index Medicus, Vol. 26, July—Dec., 1939 | | 1940 |
| St. Thomas's Hospital Reports, 2nd Series, Vol. IV. | | 1939 |
| Transactions of the American Otological Society, Vol. XXIX.z. | (1) | 1939 |

### Publications Received

From Messrs Cassell & Co. Ltd.:

*Manson's Tropical Diseases.* Edited by Philip H. Manson-Bahr, C.M.G., D.S.O., M.A., M.D., D.T.M. and H. (Cantab.), F.R.C.P. (Lond.). Eleventh Edition. Price 35s.

From General Medical Council:

*Second Addendum to the British Pharmacopoeia,* 1932.

From Messrs. H. K. Lewis & Co. Ltd.:

*Landmarks and Surface Markings of the Human Body.* By L. Bathe-Rawling, M.B., B.Ch. (Cantab.), F.R.C.S., Eighth Edition. Price 8s. 6d.

*Insect Pests.* By William Clunie Harvey, M.D., D.P.H., M.R.San.I., and Harry Hill, M.R.San.I., A.M.I.S.E., M.S.I.A. Price 10s. 6d.

*Tomography.* By J. B. McDougall, M.D. (Glas.), F.R.C.P.Ed., F.R.S.E. Price 21s.

*A Handbook of Radiography.* By John A. Ross, M.A., M.R.C.S., L.R.C.P., D.M.R.E. Price 7s. 6d.

From Messrs. E. & S. Livingstone:

*Illustrations of Bandaging and First Aid.* Compiled by Lois Oakes, S.R.N., D.N. Price 6s.

*Surgery of the Hand.* By R. M. Handfield-Jones, M.C., M.S., F.R.C.S. Price 15s.

*Anatomy. Part III. (Head and Neck).* By Charles R. Whittaker, F.R.C.S.E., F.R.S.E. Fifth Edition. Price 1s. 6d.

*Clinical Practice in Infectious Diseases.* By E. H. R. Harries, M.D. (Lond.), M.R.C.P., D.P.H., and M. Mitman, M.D. (Lond.), M.R.C.P., D.P.H., D.M.R.E. Price 17s. 6d.

*J. F. Sutherland's First Aid to Injured and Sick.* Revised and rewritten by Halliday Sutherland, M.D. Forty-second Edition. Price 6d.

*Advice to the Expectant Mother on the Care of her Health and that of her Child.* By F. J. Browne, M.D., D.Sc., F.R.C.S.E., F.R.C.O.G. Fifth Edition. Price 6d.

From Oxford University Press:

*Diseases of the Nervous System.* By W. Russell Brain, M.A., D.M. (Oxon), F.R.C.P. (Lond.). Second Edition. Price 30s.

*Cunningham's Manual of Practical Anatomy.* Revised and edited by J. C. Brash and E. B. Jamieson. Tenth Edition. Three vols. Price 15s. each.

*Groves and Brickdale's Text-book for Nurses.* By E. W. Hey Groves, M.D., B.Sc., M.S., F.R.C.S., and J. A. Nixon, C.M.G., M.D., F.R.C.P. Sixth Edition. Price 21s.

*An Introduction to Pharmacology and Therapeutics.* By J. A. Gunn, M.A., M.D., D.Sc., F.R.C.P. Sixth Edition. Price 6s.

From MESSRS. JOHN WRIGHT & SONS LTD.:

*Haemorrhoids and their Treatment.* By KASPAR BLOND, M.D. Vienna. Price 15s.

*Diseases of the Urethra and Penis.* By E. D'ARCY MCCREA, M.D., M.Ch. (Dub.), F.R.C.S.I., F.R.C.S. (Eng.). Price 21s.

*Medical Annual,* 1940. Price 20s.

*British Journal of Surgery.* No. 108. April, 1940. Price 12s. 6d.

*Ear Model for Otoscopic Practice.* Designed by A. J. WRIGHT, M.B., F.R.C.S. Price 21s.

*Pye's Surgical Handicraft.* Edited by HAMILTON BAILEY, F.R.C.S. (Eng.). Twelfth Edition. Price 21s.

### Local Medical Notes

Captain Percy K. Jenkins, Royal Army Medical Corps, has been awarded the Military Cross for gallant conduct with the B.E.F. during their evacuation from France.—*Western Daily Press.*

**Royal College of Physicians of Edinburgh.**—R. F. Barbour, M.A., M.B., Ch.B., has been admitted to the Fellowship.

EXAMINATION RESULTS.

**University of Bristol.**—Students of the University have recently passed the following examinations:—

M.B., CH.B.—*First Examination. Pass:* Grace V. Andrew, Elizabeth Bryant, Margaret J. Cox, R. W. Drewer, J. G. R. Ellis, Irene D. R. Gregory, D. H. Johnson, Margaret A. Lamb, Edith J. Lowick, D. Purnell, Dorothy M. Reece, Margaret E. J. Richards, A. D. Stewart, S. A. K. Stokes, T. Straton, W. B. Tindall, Pamela Westhead, Doris G. Virgo, Margaret M. Moncrieff, A. M. Sweeting, G. A. K. Jahans, R. A. J. Pearce.

M.B., CH.B.—*Second Examination. Pass:* Janet Baker, J. E. Beviss, Margot J. Copland, A. G. M. Davies, Mercia J. Griffiths, C. H. Gurd, Beryl M. Hinckley, J. E. G. Hope-Scott, D. E. Oliff, A. P. Radford (*a*), D. A. Sarsfield (*a*), Margaret Savile, S. S. Short (*a*, *b*), T. R. Stevens, Elizabeth N. R. Wilson. *In Group II Completing Examination:* Eileen M. Herbert, (Mrs.) Moragh Noakes, A. J. Saxton, Isabel M. M. Wilson. *In Group I only:* M. P. Bourke, J. Eskell, J. R. V. B. Gibson, W. H. N. Scawin.

M.B., CH.B.—*Final Examination (Section I). Pass:* G. T. Cribb, Winifred M. Curgenven-Robinson, Clara J. Fraser, G. H. Harvey (*c*), P. W. J. Searle.

(*a*) With Distinction in Anatomy.

(*b*) With Distinction in Physiology.

(*c*) With Distinction in Materia Medica, Pharmacy, Pharmacology, Pharmacotherapeutics and Toxicology.

M.B., CH.B. — *Final Examination. Pass (with Second Class Honours):* T. J. Butler (*d*), D. L. Bayle,y R. G. Boyd (*d*), P. Jacobs, Marjorie Organ, R. W. Orton, Patricia M. Simpson, J. J. de S. Snijman, Barbara F. Thomas (*e*), Joan Threadgold (*f*), G. H. Tovey (*f*), D. N. Walder, L. Willoughby, Winifred M. Curgenven-Robinson, Clara J. Fraser, J. K. Lewis, J. B. Tucker, G. H. Harvey, J. W. J. Newton, D. A. Squire.

B.D.S.—*First Examination.* Pass *:* H. L. Leech, Evelyn M. Pitt, I. A. Morgan.

B.D.S. — *Second Examination. Pass :* C. R. W. Birkett, Jean A. M. C. Gordon-Ralph, R. D. Hepburn.

B.D.S.—*Third Examination. Pass :* F. M. Warren (*g*), F. G. Lloyd.

L.D.S.—*First Examination. Pass :* R. D. Heald, J. J. Tittle, Eileen Rich.

L.D.S.—*Second Examination. Pass :* W. D. Arnold, W. E. C. Chaplin, H. B. Doubt, D. A. Holmes, J. Hornsby, R. E. May, B. A. Oliver, R. H. Shipway, J. B. Westlake, F. E. R. Williams, J. B. Wood.

L.D.S.—*Third Examination. Pass in Dental Mechanics and in Dental Anatomy :* G. G. Davis. *In Dental Mechanics :* G. Hellings. *In Dental Anatomy :* Patricia M. Burke, Isabella Rees.

L.D.S.—*Final Examination. Pass :* R. J. Lee, D. G. Davies, J. R. Herbert, P. Le Masurier, O. H. Minton, C. R. Sage, R. B. Stiles.

(*d*) With Distinction in Forensic Medicine and Toxicology.

(*e*) With Distinction in Surgery.

(*f*) With Distinction in Public Health.

(*g*) With Distinction in Dental Anatomy and Dental Mechanics and the Properties of Materials used in Dental Practice.

**Conjoint Board.**

L.R.C.P., M.R.C.S.—*First Examination. Pass :* B. L. Finzel (Anatomy).

L.R.C.P., M.R.C.P. — *Final Examination. Pass :* E. W. J. Townsend* (*h, j*), D. Rosser (*h*), K. A. Hall (*j, k*), C. J. R. Jacob (*k*), G. Schwizer (*j*).

L.R.C.P., M.R.C.S.—*Final Examination. Pass :* P. Jacobs (*h, j, l*), A. G. Farr (*h, j, k*), J. L. Battle (*h*), Doris Critchley (*h*), C. J. R. Jacob (*h*), K. A. Hall (*h*), J. I. Williams (*l*), J. M. Joshua (*j*), D. Rosser* (*j*).

(*h*) Medicine. (*j*) Surgery. (*k*) Midwifery. (*l*) Pathology. * Qualified.

